# Supplementary material for: Lipid Signaling Requires ROS Production to Elicit Actin Cytoskeleton Remodeling during Plant Innate Immunity
Source: Int J Mol Sci. 2022 Feb 23;23(5):2447. doi: 10.3390/ijms23052447 (PMC8910749; doi:10.3390/ijms23052447)
Supplement: Supplementary file 1 [file ijms-23-02447-s001.zip › ijms-1577256-supplementary.pdf]

Lipid Signaling Requires ROS Production to Elicit Actin Cytoskeleton Remodeling During Plant Innate Immunity

Lingyan Cao[1,4 *], Wenyi Wang[2], Weiwei Zhang[1] and Christopher J. Staiger [1,2,3 *]

[1] Department of Biological Sciences, Purdue University, West Lafayette, IN 47907, USA

[2] Department of Botany and Plant Pathology, Purdue University, West Lafayette, IN 47907, USA

[3] Center for Plant Biology, Purdue University, West Lafayette, IN 47907, USA

[4] School of Agriculture and Biology, Shanghai Jiao Tong University, Shanghai 200240, China

***** Correspondence: caolingy@sjtu.edu.cn (L.C.); staiger@purdue.edu (C.J.S.)

The following Supplementary Data are available for this article:

Figure S1. T-DNA insertion mutants of *PLD* genes.

Figure S2. Several PLD isoforms are implicated in actin remodeling.

Figure S3. Exogenous PA and H_2O_2 stimulate actin remodeling in a dose-dependent manner.

Figure S4. DPI and FIPI impair flg22-induced ROS production.

Figure S5. Time series of flg22-induced ROS production in *pldβ* mutants.

Table S1. Primers used in qRT-PCR.

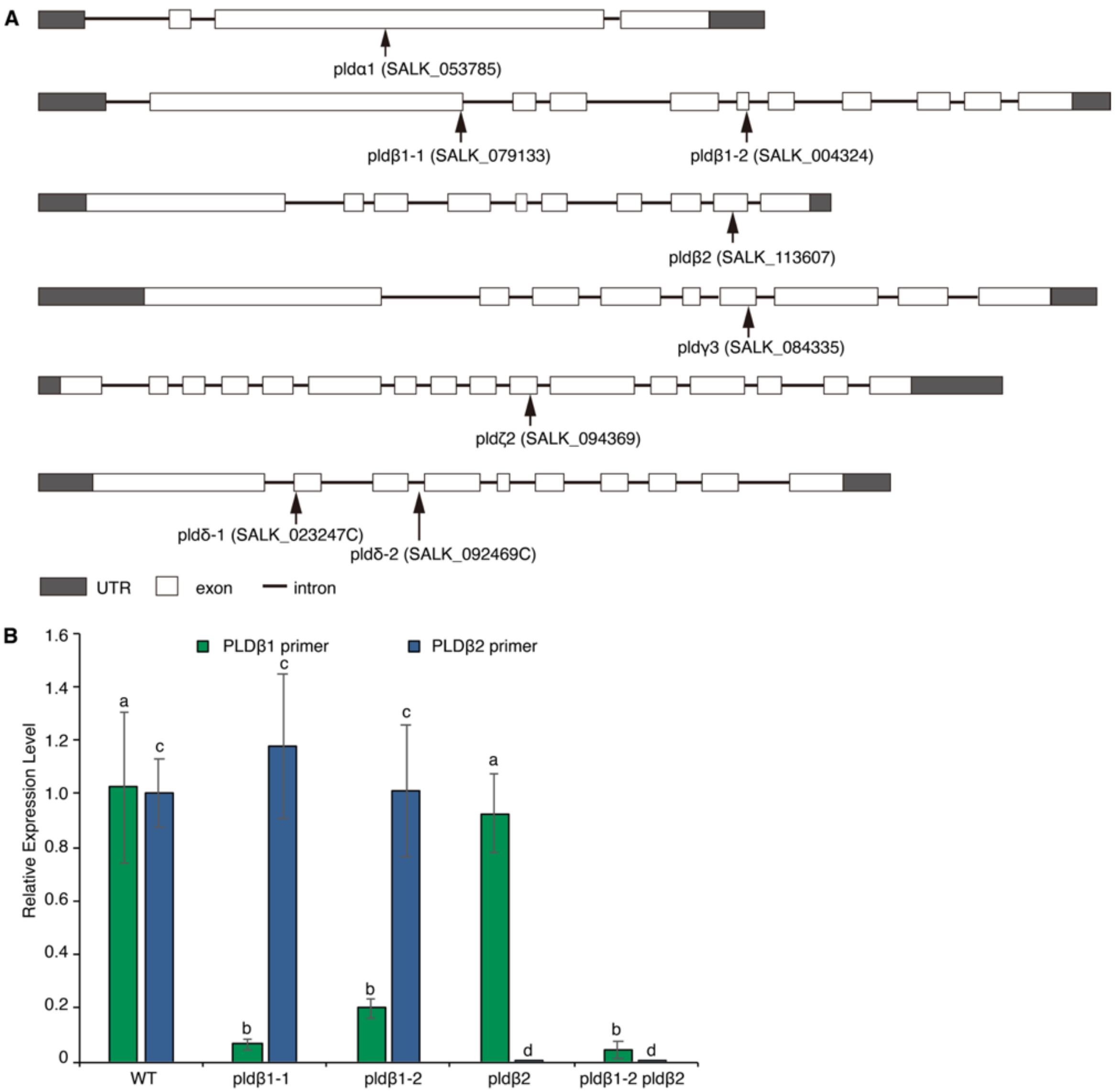

Figure S1. T-DNA insertion mutants of *PLD* genes.

(**A**) The illustrations show location of T-DNA insertions in *pld* mutant lines used in this study: *pldα1* (SALK_053785), *pldβ1-1* (SALK_079133) and *pldβ1-2* (SALK_004324), *pldβ2* (SALK_113607), *pldγ3* (SALK_084335), *pldζ2* (SALK_094369), *pldδ-1* (SALK_023247C) and *pldδ-2* (SALK_092469C). Grey boxes represent UTR (Untranslated Region); white boxes represent exons; and black lines represent introns for each *PLD* gene. These include: *PLDα1*, At5g15730; *PLDβ1*, At2g42010; *PLDβ2*, At4g00240; *PLDγ3*, At4g11840; *PLDζ2*, At3g05630; and *PLDδ*, At4g35790. (**B**) qRT-PCR to quantify the *PLDβ* expression level in *pldβ1-1, pldβ1-2, pldβ2* and *pldβ1-2 pldβ2* double mutant. *PLDβ1* gene expression was reduced in both *pldβ1-1* and *pldβ1-2* mutants, but not affected in the *pldβ2* mutant. When compared to wild type (WT), *PLDβ2* gene expression was significantly lower in *pldβ2* mutant but comparable to WT in *pldβ1* mutants. Both *PLDβ1* and *PLDβ2* gene expression were reduced in *pldβ1-2 pldβ2* double mutant. Values represent mean ± SD. Statistical analyses were conducted among data generated with the same primer set applied to different genotypes. Letters indicate significant differences among genotype using one-way ANOVA, compared with Turkey-Kramer Honestly Significant Difference (HSD) in Microsoft Excel.

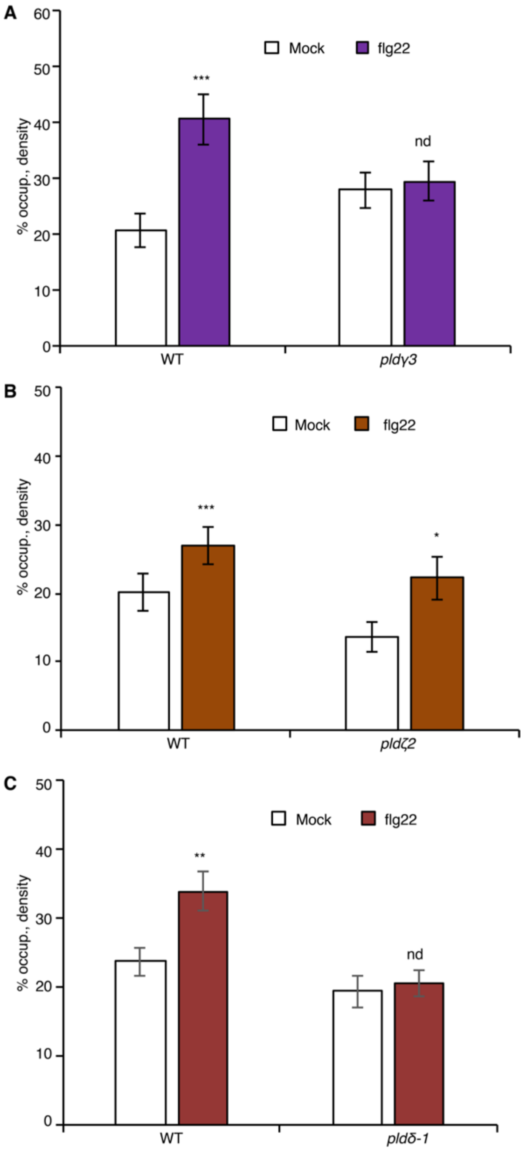

Figure S2. Several PLD isoforms are implicated in actin remodeling.

(**A**) Actin filament abundance in *pldγ3* after flg22 treatment. In epidermal pavement cells from wild-type (WT) cotyledons, 1 μM flg22 stimulated significant actin accumulation compared to mock treatment, whereas actin filament remodeling in *pldγ3* was insensitive to flg22 treatment. (**B**) The density of actin filaments increased after flg22 treatment in both WT and *pldζ2*. (**C**) Actin filament abundance in *pldδ-1* was not elicited after flg22 treatment. For ease of comparison among different genotypes, GFP-fABD2 seeds in Col-0 background were harvested at the same time with experimental mutants used for the analyses. Values given represent mean ± SE. n = 50 images from 10 seedlings per genotype and treatment. nd, no difference, *P < 0.5, **P < 0.01, ***P < 0.001, Student *t* test.

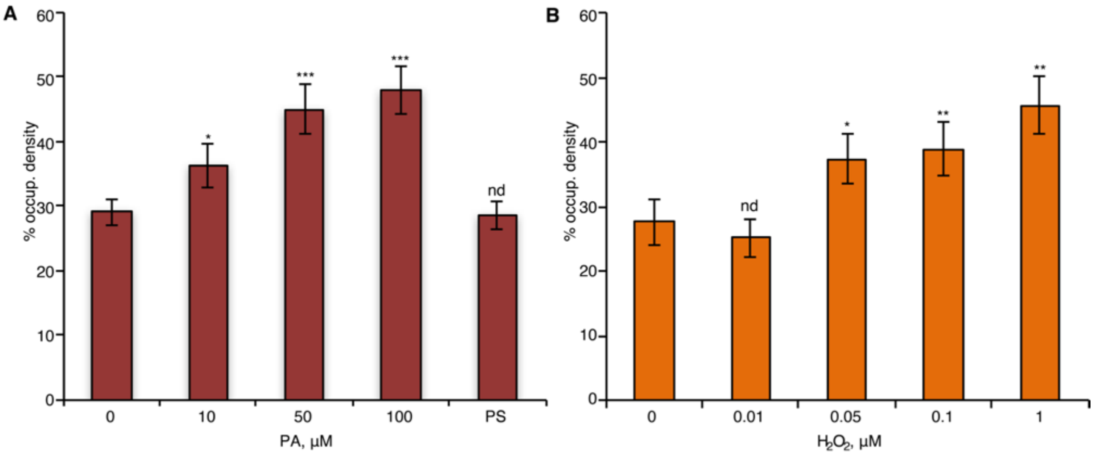

Figure S3. Exogenous PA and H_2O_2 stimulate actin remodeling in a dose-dependent manner.

(**A**) Analysis of actin filament abundance in WT cotyledons after treatment with various doses of phosphatidic acid (PA) for 30 min. Compared to mock (0 μM), the density of actin filament arrays increased after treatment with PA in a dose-dependent manner. By contrast, no actin remodeling was detected after 50 μM phosphatidylserine (PS) treatment. (**B**) Analysis of actin filament abundance in WT cotyledons treated with various doses of H_2O_2 for 20 min. The density of actin filament arrays significantly increased after treatment with ≥ 50 nM H_2O_2. Values given represent mean ± SE. n = 50 images from 10 seedlings per treatment. nd, no difference, *P < 0.05, **P < 0.01, ***P < 0.001, Student *t* test.

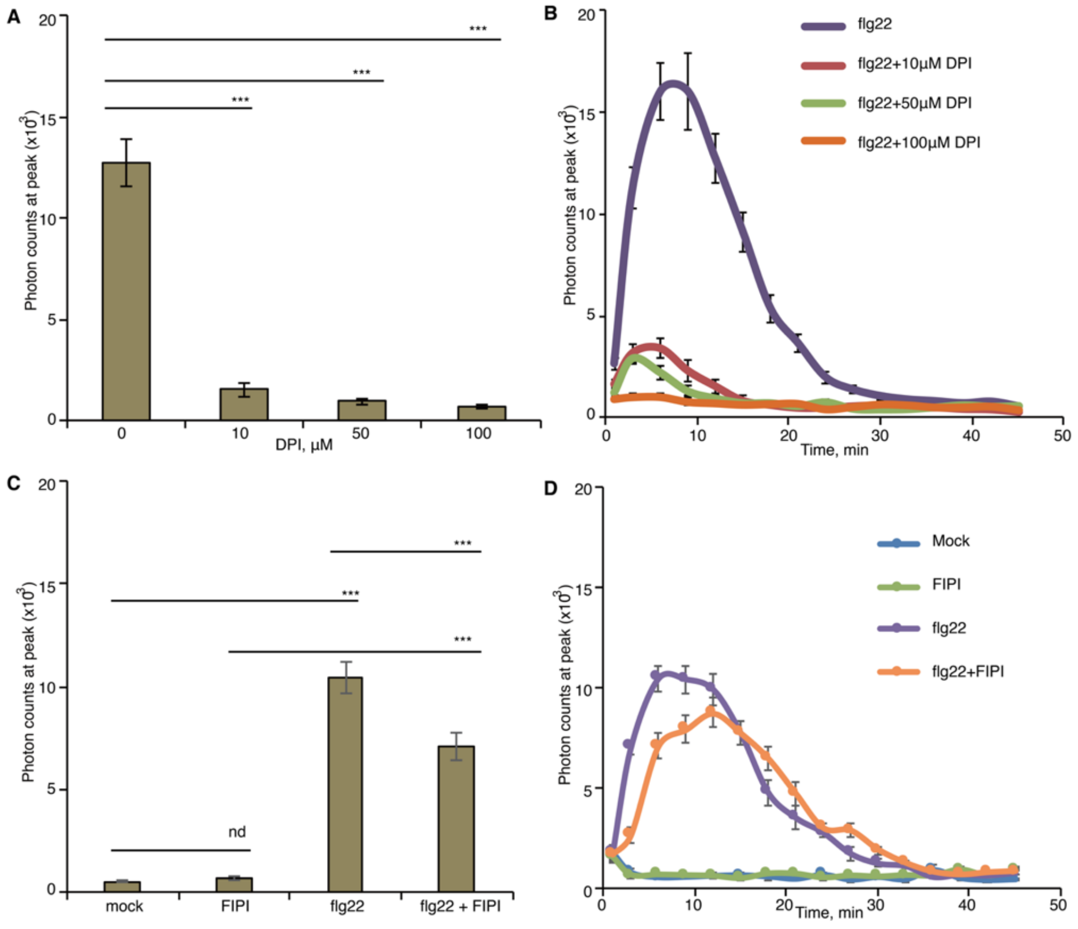

Figure S4. DPI and FIPI impair flg22-induced ROS production.

(**A,B**)**,** Apoplastic ROS production elicited by flg22 was examined in leaf disks from 4-week-old wild-type plants in the presence or absence of the NADPH oxidase inhibitor, DPI. Leaf disks were pretreated for 30 min with various concentrations of DPI prior to addition of flg22. Compared to mock, DPI inhibited ROS production in a dose-dependent manner. Values given represent mean ± SE. n = 16 repeats from 10 plants per genotype. ***P < 0.05 when compared to mock treatment, Student *t* test. (**C,D**)**,** Apoplastic ROS production in the presence of PLD activity inhibitor, FIPI. Pretreatment with FIPI for 30 min was carried out prior to addition of flg22. FIPI significantly impaired ROS production elicited by flg22. No obvious ROS was detected in the presence of FIPI alone. ***P < 0.001; nd, no difference, ndP ≥ 0.05; Student *t* test.

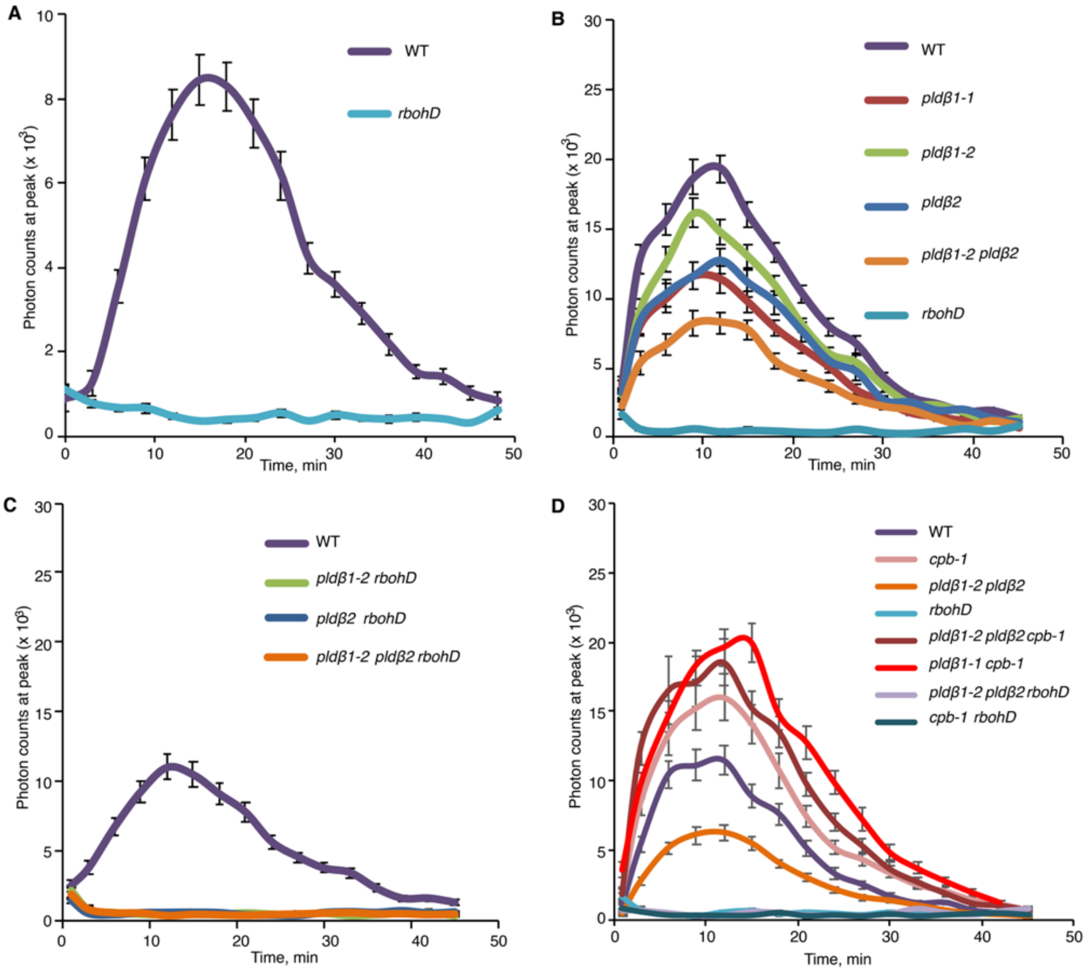

Figure S5. Time series of flg22-induced ROS production in *pldβ* mutants.

(**A**) Apoplastic ROS production elicited by flg22 was examined in cotyledons from 8-day-old wild-type (WT) and *rbohD* plants. A strong signal was detected in WT around 12–15 min after flg22 treatment. (**B**) ROS production after flg22 treatment was monitored in leaf disks from 4-week-old plants. In *pldβ1-1*, *pldβ1-2*, *pldβ2*, and *pldβ1-2 pldβ2* ROS production was attenuated compared to WT. *rbohD* was used as a negative control. (**C**) Apoplastic ROS production in *pldβ1-2 rbohD*, *pldβ2 rbohD*, and *pldβ1-2 pldβ2 rbohD*. Removal of RBOHD in the *pldβ* mutants blocked flg22-induced ROS production. (**D**) Apoplastic ROS production in *pldβ1-2 pldβ2 cpb-1* and *pldβ1-1cpb-1*. ROS production in *pldβ1-2 pldβ2 cpb-1* and *pldβ1-1 cpb-1* was higher than that in WT and *cpb-1* alone. *pldβ1-2 pldβ2, pldβ1-2 pldβ2 rbohD,* and *rbohD* were used as controls. Values given represent mean ± SE. n = 16 repeats from 10 plants per genotype.

Table S1. Primers used in qRT-PCR.

	**Sequence (5′– 3′)**	**Note**
PLDβ1-F	GAGACAGGGCTTGAAGGAGCA	Forward
PLDβ1-R	CTTCTGCTTTTCCGACTCAACGC	Reverse
PLDβ2-F	CTAGATGATTGCTTTGTGGAGC	Forward
PLDβ2-R	CACTTCCTCACTCCTAAACTGT	Reverse
UBC-F	CTGCGACTCAGGGAATCTTCTAA	Forward
UBC-R	TTGTGCCATTGAATTGAACCC	Reverse
